# Gut Microbiota in a Rat Oral Sensitization Model: Effect of a Cocoa-Enriched Diet

**DOI:** 10.1155/2017/7417505

**Published:** 2017-01-25

**Authors:** Mariona Camps-Bossacoma, Francisco J. Pérez-Cano, Àngels Franch, Margarida Castell

**Affiliations:** ^1^Section of Physiology, Department of Biochemistry and Physiology, Faculty of Pharmacy and Food Science, University of Barcelona, 08028 Barcelona, Spain; ^2^Nutrition and Food Safety Research Institute (INSA-UB), 08921 Santa Coloma de Gramenet, Spain

## Abstract

Increasing evidence is emerging suggesting a relation between dietary compounds, microbiota, and the susceptibility to allergic diseases, particularly food allergy. Cocoa, a source of antioxidant polyphenols, has shown effects on gut microbiota and the ability to promote tolerance in an oral sensitization model. Taking these facts into consideration, the aim of the present study was to establish the influence of an oral sensitization model, both alone and together with a cocoa-enriched diet, on gut microbiota. Lewis rats were orally sensitized and fed with either a standard or 10% cocoa diet. Faecal microbiota was analysed through metagenomics study. Intestinal IgA concentration was also determined. Oral sensitization produced few changes in intestinal microbiota, but in those rats fed a cocoa diet significant modifications appeared. Decreased bacteria from the Firmicutes and Proteobacteria phyla and a higher percentage of bacteria belonging to the Tenericutes and Cyanobacteria phyla were observed. In conclusion, a cocoa diet is able to modify the microbiota bacterial pattern in orally sensitized animals. As cocoa inhibits the synthesis of specific antibodies and also intestinal IgA, those changes in microbiota pattern, particularly those of the Proteobacteria phylum, might be partially responsible for the tolerogenic effect of cocoa.

## 1. Introduction

Apart from its nutritional role, food intake influences intestinal tissue and increasing evidence exists regarding the interaction among diet, immune system, and microbiota. Food intake determines the composition of microbiota and the function of gut-associated lymphoid tissue (GALT). These last two factors are also closely related, so a vast number of diets alter bacteria composition, thereby affecting immune homeostasis, and vice versa [1]. Gut microbiota, which in the adult human tract contains more than 100 trillion bacteria and more than 150 times the number of genes compared to the host genome [2, 3], provides several benefits to the intestinal immune system. Therefore, intestinal bacteria are critical for the regulation of the immune system and barrier function [3] and play an important role in the development of both innate and acquired response, promoting the expansion of B and T cells in Peyer's patches and mesenteric lymph nodes [4]. The intestinal immune system characteristically produces antibodies belonging to the immunoglobulin A (IgA) isotype. IgA is the most abundant immunoglobulin in the body and is considered to be the first line of defence in protecting the intestine against ingested pathogens [5].

Among the most prevalent diseases related to a faulty immune system function are allergic diseases. In Western countries, the prevalence of allergic diseases, including food allergy, is increasing and has become a major public health concern [3]. An allergic response generally occurs when antigen-presenting cells present the antigen to T helper (Th) lymphocytes, which once activated, proliferate and turn mainly into Th2 effector cells, secreting their characteristic cytokine pattern [6]. Nowadays, an association between changes in microbiota and high susceptibility to allergy is recognized [7, 8]. Therefore, the hygiene hypothesis suggests that the later the microbial exposure, the greater the prevalence of allergic diseases [9]. It has been reported that germ-free mice undergo an increase in the development of oral allergic sensitization, which is the first step in allergy disease [10]. Therefore, microbiota is important for the induction of oral tolerance that protects from food allergies [11]. In particular, the administration of a main human bacterium,* Bacteroides fragilis *[12], and a mixture of* Clostridia* strains [13] can increase the development of regulatory T (Treg) cells and, therefore, inhibit the development of oral allergy.

As previously stated, food influences microbiota and the intestinal immune system. Among the bioactive components with this recognized action are flavonoids, a kind of polyphenols, which, besides their antioxidant properties, modulate bacterial growth and composition and which influence toll-like receptor (TLR) activation as well as inflammatory and immune response activation [14]. Oligomeric and polymeric polyphenols can reach the colon [15], and published data in human and in vitro and in vivo animal models indicate their role in changing microbiota composition (reviewed in [16, 17]). In addition, some flavonoids have shown their antiallergic potential (reviewed in [18]), a fact that could be related to their impact on the composition of gut microbiota [19]. One food relatively rich in flavonoids is cocoa, which also contains carbohydrates, proteins, lipids, fibre, minerals, and methylxanthines. A few studies have been published that discuss the effect of cocoa on gut microbiota. Feeding of 10% cocoa diet for 6 weeks in Wistar rats decreased the proportion of* Bacteroides*, the* Staphylococcus* genus, and the* Clostridium histolyticum* subgroup [20]. Another study in which three different amounts of cocoa polyphenols were given to the same rat strain for 4 weeks described the age-dependent inhibition of the growth of* Staphylococcus*,* Streptococcus*,* Clostridium histolyticum,* and* Clostridium perfringens,* which was partially attributed to their polyphenol content [21].

Recently we demonstrated the tolerogenic effect of a 10% cocoa diet on a rat oral sensitization model [22]. These results led us to ascertain whether a cocoa diet may exert its effects, at least partially, by influencing the microbiota composition in this rat oral sensitization model [22, 23]. Taking all these facts into consideration, the aim of the present study was to establish the influence of an oral sensitization model, both alone and together with a cocoa-enriched diet, on gut microbiota.

## 2. Materials and Methods

### 2.1. Animals and Diets

Female Lewis rats were obtained from Janvier (Saint-Berthevin Cedex, France) and housed in cages under controlled temperature and humidity in a 12 h light-12 h dark cycle in the Faculty of Pharmacy and Food Sciences' animal facility. The procedures used in the current study were approved by the Ethics Committee for Animal Experimentation of the University of Barcelona (CEEA/UB ref. 5988).

Three-week-old rats were randomly distributed into three groups (*n* = 6 each) according to the diet and the oral sensitization procedure: the reference (RF) group (standard diet and no oral sensitization), the ovalbumin (OVA) group (standard diet and oral OVA sensitization), and the OVA/C group (10% cocoa diet and oral OVA sensitization). The diet lasted for four weeks during which the animals had free access to food and water. AIN-93M (from Harlan Teklad, Madison, Wisconsin, USA) formula was used as the standard diet and a 10% cocoa diet was produced through modification of the standard formula, adjusting the amounts of carbohydrates, proteins, lipids, and fibre in accordance with the 10% of cocoa powder (from Idilia Foods SL, formerly Nutrexpa SL, Barcelona, Spain) as described previously [22]. The diets were isoenergetic and had the same proportion of macronutrients and micronutrients. The cocoa diet contained 40.18 mg/g of total polyphenols (expressed as catechin) determined according to Folin-Ciocalteu method.

### 2.2. Oral Sensitization

Rat oral sensitization was induced as previously described [22]. In brief, rats from sensitized groups received orally 50 mg of OVA (grade V; Sigma-Aldrich, Madrid, Spain) with 30 *μ*g of cholera toxin (CT; Sigma-Aldrich) as adjuvant in 1 mL of distilled water by oral gavage, three times per week for three weeks (on days 0, 2, 4, 7, 9, 11, 14, 16, 18, and 21). However, the RF group received just 1 mL of the vehicle with the same frequency of administration. This procedure is able to induce the synthesis of specific anti-OVA antibodies [22, 23].

### 2.3. Sample Collection and Processing

Faecal samples were collected before oral sensitization and once per week afterwards (days 0, 7, 14, 21, and 28) and processed in order to obtain faecal homogenates as previously described [24]. Briefly, faecal samples were dried and weighed, and phosphate-buffered saline (PBS, pH 7.2) was added to obtain a final concentration of 20 mg/mL. Immediately, the mix was homogenized with a Polytron® (Kinematica, Lucerne, Switzerland) and centrifuged, and supernatants were frozen at −20°C until total IgA quantification. Moreover, fresh faecal samples from day 23 were, on the one hand, weighed, dried for 5 h at 37°C, and weighed again in order to determine the percentage of humidity as an indicator of faecal consistency and, on the other hand, used for faecal pH determination using a surface electrode (Crison Instruments, SA, Barcelona, Spain).

### 2.4. Quantification of Intestinal IgA

IgA from faecal homogenates was quantified using a sandwich enzyme-linked immunoabsorbent assay (ELISA) technique with a Rat IgA ELISA Quantification Set (E110-102) from Bethyl Laboratories (Montgomery, TX, USA). Briefly, 96-well plates (Nunc MaxiSorp®, Wiesbaden, Germany) were coated with 2 *μ*g/mL of the capture antibody in carbonate buffer (pH 9.6). After blocking, the standard and the samples were incubated. Finally, an adequate dilution of the peroxidase-conjugated detection antibody was added and, after washing, an* o*-phenylenediaminedihydrochloride-H_2_O_2_ (OPD-H_2_O_2_) (Sigma-Aldrich) solution was added. Absorbance was measured in a microplate photometer. Data were interpolated by Multiskan Ascent v2.6 software (Thermo Fisher Scientific SLU, Barcelona, Spain) according to the concentration of the standard.

### 2.5. Faecal Metagenomic Analysis

DNA from three representative faecal samples from each group from the 28th day of the experimental design was extracted using a FastDNA® SPIN kit (MP Biomedicals, Solon, Ohio, USA) according to the manufacturer's instructions. An Ion 16S™ Metagenomics kit (Life Technologies, Madrid, Spain) was used for the metagenomic study carried out by Bioarray Genetic Diagnosis (Bioarray, Alicante, Spain).

After confirming that all DNA samples had good levels of concentration, purity, and integrity, a massive sequentiation was carried out with the platforms QIIME v1.8.0 and USEARCH v.7.0.1090. In order to assign the taxonomy, the different sequences with 97% similarity were assembled into operational taxonomic units (OTUs) using the data base GreenGenes v13_8 with the UCLUST method.

### 2.6. Statistical Analysis

Statistical analysis was performed using IBM's Statistical Package for Social Sciences program (SPSS, version 22.0, Chicago, IL, USA). Differences were considered statistically significant when *p* < 0.05.

In order to determine equality of variance and normal distribution, the Levene and Kolmogorov-Smirnov tests, respectively, were carried out. One-way ANOVA and Bonferroni's post hoc test were performed on the results with equality of variance and normal distribution. The nonparametric Mann–Whitney* U* test was performed on the data that did not have equality of variance and/or normal distribution.

Bivariate Pearson correlation was used to determine whether an association exists between intestinal IgA concentration and either relative abundance, absolute abundance, or the number of detected bacterial species.

## 3. Results

### 3.1. Effect of Cocoa on Faecal pH and Humidity in Orally Sensitized Rats

**Figure 1:**
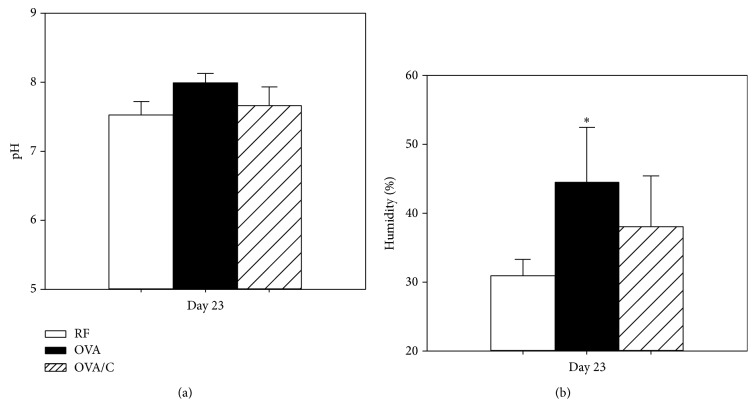
Faecal pH and humidity at day 23 of the study. Values are expressed as mean ± standard error (*n* = 6). Percentage of humidity was calculated as follows: ((initial faecal weight − dry faecal weight)/initial faecal weight) × 100. Dry faecal weight was considered after 5 h at 37°C. Statistical differences: ^*∗*^*p* < 0.05 versus RF group (Mann–Whitney* U* test).

Faecal pH and humidity were determined on day 23 of the study (Figure 1). The RF group had a faecal pH average of 7.52 and no differences were detected due to the oral sensitization or the cocoa diet (Figure 1(a)). In contrast, the orally sensitized group showed a higher faecal humidity (Figure 1(b)), exhibiting more water content than the RF group, whereas no significant differences with respect to the OVA/C group were found.

### 3.2. Effect of Cocoa on the Intestinal IgA Concentration in Orally Sensitized Rats

**Figure 2:**
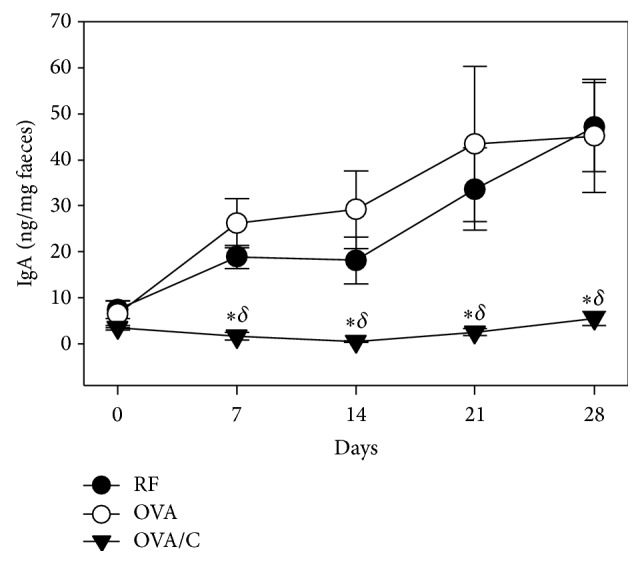
Intestinal IgA concentration during the study. Values are expressed as mean ± standard error (*n* = 6). Statistical differences: ^*∗*^*p* < 0.05 versus RF group and ^*δ*^*p* < 0.05 versus OVA group (Mann–Whitney* U* test).

Faecal IgA determination revealed that the animals fed the standard diet, whether or not they received the oral sensitization, increased IgA concentration during the study. However, this time-dependent increase was inhibited from day 7 due to the 10% cocoa diet (Figure 2).

### 3.3. Effect of Cocoa on Gut Metagenome in Orally Sensitized Rats

#### 3.3.1. Quantitative Metagenomic Study

**Figure 3:**
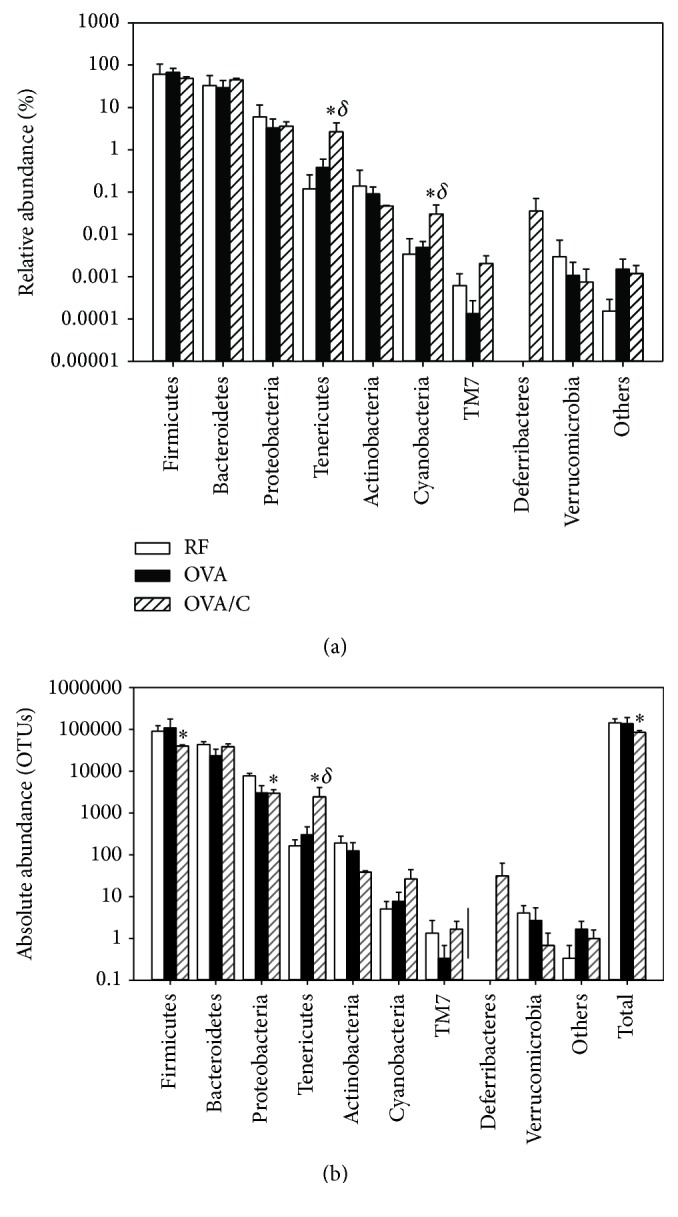
Bacteria phyla on faecal gut microbiota. (a) Relative abundance (%) and (b) absolute abundance in the groups of study. Values are expressed as mean ± standard error (*n* = 3). OTUs: operational taxonomic units. Statistical difference: ^*∗*^*p* < 0.05 versus RF group and ^*δ*^*p* < 0.05 versus OVA group (Mann–Whitney* U* test).

As shown in Figure 3(a), from the total microbiota detected in reference rats, 61% of the bacteria belonged to the Firmicutes phylum, 33% to Bacteroidetes, 6% to Proteobacteria, and less than 1% to the Tenericutes, Actinobacteria, Cyanobacteria, Verrucomicrobia, and TM7 phyla. From these phyla, no significant differences were found in the OVA group with respect to the RF group. However, those sensitized rats fed a cocoa-enriched diet (OVA/C group) showed a higher proportion of bacteria belonging to the Tenericutes and Cyanobacteria phyla compared to those from the RF and OVA groups.

The study of absolute bacterial abundance also revealed significant changes in animals from the OVA/C group (Figure 3(b)). The orally sensitized group fed a cocoa diet had a lower amount of total bacteria compared to the RF group, which could be attributed to a reduction in the number of Firmicutes and Proteobacteria. However, a higher proportion of Tenericutes with respect to the RF and OVA groups was observed.

Furthermore, a deeper analysis revealed significant changes in the relative abundance inside each phylum (Table 1). Oral sensitization decreased the proportion of bacteria belonging to the Erysipelotrichales order (Firmicutes phylum) in animals fed with either a standard or cocoa diet. Moreover, the cocoa diet lowered the proportion of bacteria from the RF32 order belonging to the Alphaproteobacteria class (Proteobacteria phylum). However, this diet favoured the presence of Chloroplast class (Cyanobacteria phylum), particularly the Streptophyta order, and increased the percentage of the Mollicutes class, specifically the RF39 order. 

Table 2 shows the changes found at family, genus, and species level. OVA sensitization with both standard and cocoa diets decreased the relative abundance of an unknown species of the Bacteroidales order,* Clostridium metallolevans, *and* Allobaculum* sp. Moreover, animals from the OVA/C group had lower percentages of* Ruminococcus flavefaciens*, one species belonging to the Erysipelotrichaceae family,* Holdemania* sp., and one specific species of the RF32 order, compared to the RF and/or OVA groups. On the other hand, the proportion of three species of the Prevotellaceae family, a species of the Streptophyta order,* Lactobacillus reuteri*,* Anaerostipes* sp., a species of the Mogibacteriaceae and Erysipelotrichaceae families, and a species of the Mollicutes class had a higher percentage in the cocoa-fed animals (OVA/C group) with respect to the RF and/or OVA groups.

#### 3.3.2. Qualitative Metagenomic Study

**Figure 4:**
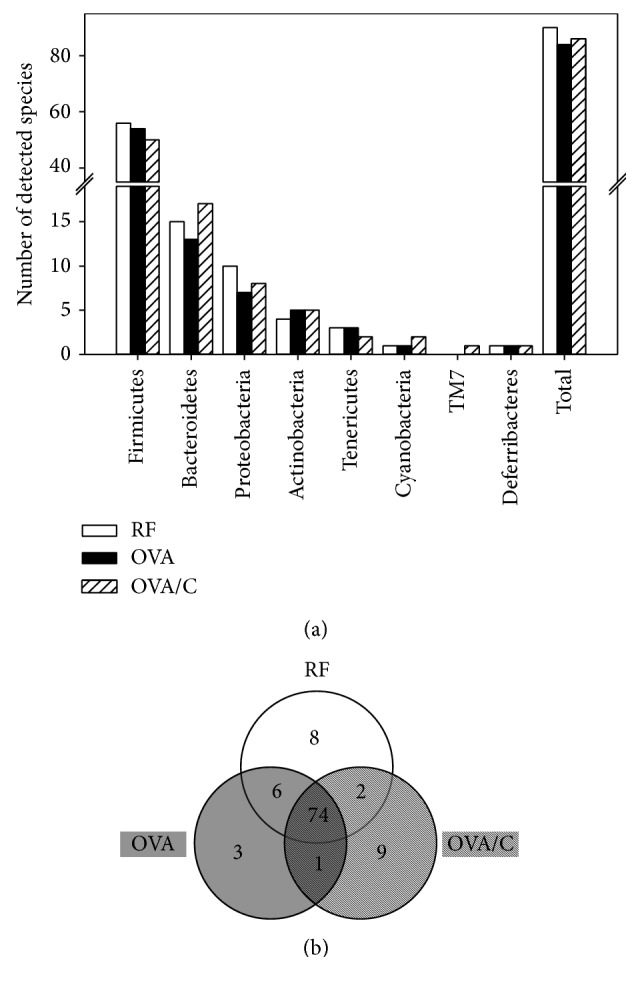
Number of detected species in faeces from each studied group. (a) Data is expressed as the total number of species detected. A species was detected if present in at least two of the three rats of each group. (b) Venn diagram of the different detected species. The diagram shows the absolute number of detected species that belong to each group, the detected species in common between each pair of groups, and, in the centre, the detected species in common among all three groups.

The metagenomic analysis also provides us with qualitative information about the gut bacterial pattern. The number of species present in at least two of the three rats from each group was counted. A total of 90 species were detected in the RF group, 84 species in the OVA group and 86 species in the OVA/C group. The number of different species classified into the different phyla is shown in Figure 4(a). For all animals, the highest bacteria richness was found in the Firmicutes, Bacteroidetes, and Proteobacteria phyla.

In order to establish the differences among the bacteria species found in each group, a Venn diagram was plotted (Figure 4(b)). Out of all the faecal detected species, 74 were present in the three studied groups. However, some modifications were detected due to the oral sensitization, the cocoa diet, or both. In reference conditions, eight different species were unique in the RF group, meaning that these species disappeared due to the oral sensitization (OVA and OVA/C groups). Four of these belonged to the Firmicutes phylum, three to the Proteobacteria phylum, and one to the Verrucomicrobia phylum (Table 3). Three species from the Firmicutes phylum were included in the* Staphylococcus* genus (e.g.,* S. equorum*), whereas the other one corresponded to* Clostridium metallolevans*. With regard to the Proteobacteria phylum, the three species that disappeared due to the oral sensitization procedure belonged to either the Alphaproteobacteria class (Rhodospirillales order), the Deltaproteobacteria class (*Spirobacillales* order), or the Gammaproteobacteria class (Vibrionales order). In addition,* Akkermansia muciniphila*, from the Verrucomicrobia phylum, was not found in orally sensitized groups.

In the OVA group, four new species were detected with respect to the RF animals. Three of them were only found in sensitized animals fed a standard diet and one was also present after the cocoa diet. From these new species, two belonged to the Firmicutes phylum, one to the Tenericutes phylum, and one to the Actinobacteria phylum. The Firmicutes phylum species included* Bacillus* and* Christensenella* genera, the Tenericutes phylum included the* Anaeroplasma* genus (Table 3), and the Actinobacteria phylum species also found in the OVA/C group was* Bifidobacterium pseudolongum* (Table 4).

With regard to the sensitized group fed a cocoa diet, nine different species were found with respect to the RF and OVA groups (Table 3). Two species belonged to the Bacteroidetes phylum, one of those being* Prevotella copri*. From the Cyanobacteria phylum, one species from the Streptophyta order was present. As regards the Firmicutes phylum, three species from the Clostridiales order were detected, belonging to the Dehalobacteriaceae, Lachnospiraceae and Veillonellaceae families. Moreover, two new species appeared from the Proteobacteria phylum (*Ralstonia* sp. and* Desulfovibrio* sp.), and a new TM7 bacterium was also found in the OVA/C group.

It is worth noting that two bacterial species were not found in the OVA group but were present in both the RF and OVA/C groups, suggesting that the cocoa diet failed to eliminate these species due to the oral sensitization. These bacteria belonged to the Bacteroidetes phylum,* Bacteroides uniformis* and* Prevotella* sp. in particular (Table 4). Moreover, six species present in both the RF and OVA groups disappeared with the cocoa diet: five of those belonged to the Firmicutes phylum, in particular the* Clostridia* (e.g.,* Clostridium perfringens, Blautia producta, *and* Epulopiscium *sp.) and Erysipelotrichi (*Coprobacillus* sp.) classes and one to the Proteobacteria phylum, specifically* Desulfovibrio* sp. (Table 4).

### 3.4. Intestinal IgA and Microbiota Associations

In order to determine whether microbiota was associated with intestinal IgA, a linear regression analysis was performed between IgA values and data from relative and absolute abundance of bacteria and the number of detected species of each phylum. As shown in Table 5, although no significant correlation for total values was found in any of the above variables, a strong positive correlation between intestinal IgA levels and Proteobacteria phylum relative abundance was found. Apart from that, no significant correlations were seen between the relative abundance, absolute abundance, or the number of detected species from each phylum and intestinal IgA concentration.

## 4. Discussion

**Figure 5:**
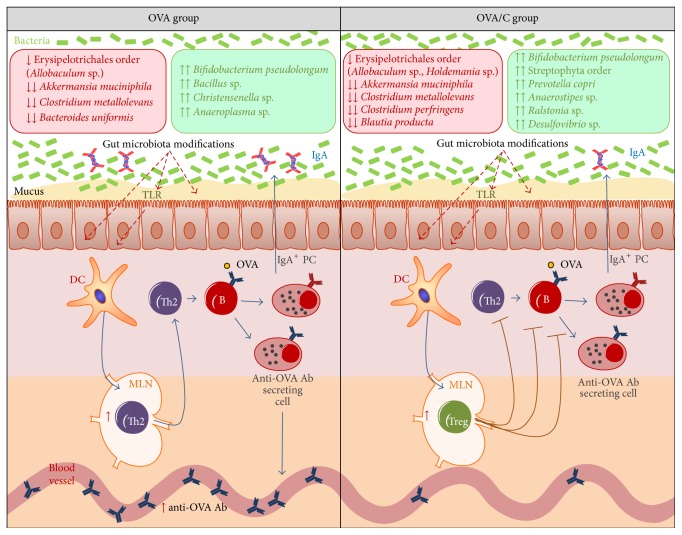
Summary of the changes on gut microbiota on a rat oral sensitization model fed either a standard diet or a 10% cocoa diet and its possible effects on the immune system. Discontinuous red arrows show the possible mechanism induced by the altered gut microbiota on the immune system. ↓ = decrease on the relative or absolute abundance. ↑ = increase on the relative or absolute abundance. ↓↓ = disappearance of the species. ↑↑ = appearance of the species. Ab: antibody; B: B lymphocyte; DC: dendritic cell; MLN: mesenteric lymph node; OVA: ovalbumin; PC: plasma cells; Th: T helper; TLR: toll-like receptor; Treg: T regulatory lymphocyte.

In healthy conditions, cocoa components are able to produce some modifications in both human and rat intestinal microbiota as previously demonstrated by using FISH technique [20, 25]. The current study, by means of a metagenomic approach, was able to go more deeply into establishing the effect of a cocoa diet and also an oral sensitization procedure on rat gut microbiota. In this study we describe microbiota changes appearing in orally sensitized animals fed both a standard and cocoa diet (Figure 5), which means that the changes must be due to oral sensitization; moreover, we found microbiota alterations only in the orally sensitized animals fed the standard diet meaning that the cocoa diet prevented such effects induced by OVA and CT; and finally we observed microbiota modifications only in animals fed cocoa, which suggest these were mainly due to cocoa diet.

The oral sensitization did not induce any significant change at the phyla level. These results do not match studies demonstrating alterations in microbiota due to food allergy, such as increases in the abundance of bacteria from the Firmicutes phylum and decreases in those belonging to the Bacteroidetes, Proteobacteria, and Actinobacteria phyla [26, 27]. Although no significant modifications at phyla level were observed in our sensitization model, in the Firmicutes phylum, the OVA plus CT administration in both diets decreased the relative abundance of bacteria belonging to the Erysipelotrichales order, which is in line with data obtained after the oral sensitization of Il4raF709 transgenic mice [28]. A deeper analysis revealed that the oral sensitization reduced the relative abundance of the Erysipelotrichaceae family and the* Allobaculum* genus. It must be noted that the Erysipelotrichi class, and particularly the* Allobaculum* genus, have been associated with a better mucus layer in the colon [29], suggesting that their decrease reflects the alteration of the mucus layer by oral sensitization that could not be prevented by the cocoa diet. On the other hand, some qualitative changes in the microbiota composition appeared due to sensitization: new bacteria colonized the damaged mucosa (four new species) and some others could not resist the new environment (ten species disappeared), which also suggests lower diversity, which is in accordance with what happened in children with eczema [30]. With regard to the bacteria species that were not found in orally sensitized animals, the absence of* Akkermansia muciniphila*, from the Verrucomicrobia phylum, is of particular interest. This Gram-negative anaerobic bacterium plays a role in host immune response and the restoration of mucus layer thickness and mucus production, secreting important proteins to the mucus [31], and is decreased in many diseases, such as intestinal disorders, inflammatory diseases, obesity, and type 2 diabetes [32].* A. muciniphila* has recently been proposed as a new functional microbe with probiotic properties [33] and its absence in orally sensitized animals found here affirms its protective role.

On the other hand, the altered intestinal environment induced by the oral sensitization procedure in both standard and cocoa-fed animals led to the new colonization of the* Bifidobacterium pseudolongum*, which belongs to the less predominant* Bifidobacteria* in infants, representing in those around 2% of the* Bifidobacterium* count [34]. It has been described that* B. pseudolongum* increased differentially in rats fed two kinds of prebiotic diets [35]. Therefore, we suggest that the sensitization procedure may affect rat's diet components biodisponibility and lead to a significant difference in the gut environment that selectively enhances this particular bacteria's growth. In addition, our results are in line with the absence of these bacteria in 18-week-old healthy Wistar rats and with their abundance in animals under two other dysbiotic conditions: exercise and obesity [36].

Considering the effect of a cocoa diet on orally sensitized animals, a vast number of modifications were seen with respect to animals fed standard diets both in healthy and in sensitized conditions. The cocoa diet in this sensitization model decreased the total bacterial count similarly to healthy rats fed cocoa containing 2% polyphenols [21]. Specifically, the cocoa diet favoured the reduction of the absolute abundance of the Firmicutes and Proteobacteria phyla, whereas more Tenericutes were observed. Moreover higher relative abundance of Tenericutes and Cyanobacteria spp. was found. With regard to the increase in Cyanobacteria, this was accompanied by the appearance of bacteria belonging to the Streptophyta order in rats fed cocoa, but not in rats fed a standard diet. As far as we know, the role of such bacteria in the intestinal microbiota remains to be elucidated, and further studies must be carried out to establish the relationship between these specific bacteria and the tolerance effects of cocoa. On the other hand, the increase in the Tenericutes phylum, partially due to bacteria belonging to RF39 order (Mollicutes class), together with the appearance of a species belonging to the TM7 phylum, could be an adaptation to the fibre content of the cocoa diet because both phyla have been associated with crude fibre digestibility in pigs [37]. In addition, bacteria from the Tenericutes phylum could provide some beneficial effects in the intestinal integrity because lower counts of these bacteria were found in intestinal inflammation induced by dextran sodium sulphate [38].

Although a cocoa diet did not influence the absolute abundance of the Bacteroidetes phylum, it increased some families from this phylum. Thus, orally sensitized rats fed a coca diet increased the relative abundance of the* Prevotella* genus and* Bacteroides uniformis. *These results could be associated with cocoa's polyphenol content since they are found elevated in humans who consume red wine polyphenols daily [39], and* Prevotella* is more common in people consuming a plant-rich diet [40]. Moreover,* B. uniformis* is able to secrete antimicrobial proteins that antagonize strains of the same species [41], which could explain why the cocoa diet decreased other Bacteroidales bacteria. With regard to the* Prevotella* genus,* P. copri*, which has been associated with improvements of glucose tolerance in mice [40], appeared in orally sensitized rats fed cocoa. This could partially explain the effect on glucose tolerance by a similar cocoa diet on Zucker diabetic fatty rats [42].

The cocoa diet also influenced the bacterial pattern of the Firmicutes phylum. The cocoa diet decreased the absolute counts of these bacteria, which was accompanied not only by decreases but also increases in some particular families of bacteria. In animals fed cocoa there was a higher proportion of* Lactobacillus reuteri*, beneficial bacteria that when administered orally in humans induced the expression of proinflammatory Th1 cytokines but not the anti-inflammatory Th2 ones [43]. This effect, which is in line with the attenuation of Th2 responses by cocoa [44], might contribute to the prevention of sensitization observed here and demonstrated with an oral treatment with live* L. reuteri* in a model of airway allergy [45]. On the other hand, the cocoa diet decreased the counts of* Ruminococcus flavefaciens* and some bacteria of the Erysipelotrichaceae family, although an unknown species from the latter family increased significantly.* R. flavefaciens* are bacteria able to degrade plant cell-wall polysaccharide [46], but they were found to be decreased after a particular condensed tannins diet in bovine rumen, which suggests again that cocoa components can modify the bacterial growth pattern in the gut [47].

On the other hand, as previously described in the same oral sensitization procedure, a cocoa diet is able to induce oral tolerance and inhibit the synthesis of specific anti-OVA antibodies [22]. These effects were accompanied by an increase in TCR*γδ* cells and CD103+CD8+ cells in mesenteric lymph nodes from cocoa-fed animals [22], cells associated with a regulatory function. In addition, as gut microbiota enhance Treg development and function [48], changes effected in the gut microbiota by cocoa could also contribute to oral tolerance throughout Treg cells (Figure 5).

Finally, here we found that both groups of rats fed a standard diet produced increasing amounts of intestinal IgA during the study period. On the other hand, the oral sensitization increased faecal water content in line with results obtained by using CT as an oral adjuvant [49]. The cocoa diet partially avoided the increase in faecal humidity and also reduced the time-dependent increase in intestinal IgA. This last effect is in line with previous results obtained in healthy conditions [50] and also confirms those derived from gut lavage and serum in the same rat oral sensitization procedure [22]. It is worth noting the correlation between intestinal IgA and the Proteobacteria phylum, whereby the more relative abundance of Proteobacteria, the higher IgA levels. These results agree with suggestions that bacteria from the Proteobacteria phylum are the main inducers of IgA by B cells [51]. B cells are responsible for the regulation of commensal bacteria producing IgA [52], so the more relative abundance of Proteobacteria could activate B cells for IgA production, evidencing higher levels of these mucosal antibodies. Previous studies have associated the effect of a 10% cocoa diet on the reduction of IgA with gene expression modifications of several genes involved in the differentiation and maturation of B cells [53, 54]. In this sense, IL-6 gene expression is reduced by the cocoa diet [53], which could reflect a lower IL-6 secretion by dendritic cells, thus partially explaining the possible dendritic cell involvement in that process. Anyway, our results allow us to suggest that oral tolerance can be achieved with low levels of IgA, although this antibody has been associated with this kind of unresponsiveness [55].

## 5. Conclusions

This study demonstrates that a cocoa diet, by means of its content of antioxidant polyphenols, fibre, or other bioactive compounds, such as theobromine, is able to modify the microbiota bacterial pattern in orally sensitized animals. As cocoa inhibits the synthesis of specific antibodies and also the production of intestinal IgA, those changes in microbiota composition, particularly those of the Proteobacteria phylum, might be partially responsible for this tolerogenic effect of cocoa.

## Figures and Tables

**Table 1:** Bacteria order on faecal gut microbiota. Relative abundance (%) of the statistically different significant orders of bacteria. Values are expressed as mean ± standard error (*n* = 3). Statistical difference: ^*∗*^*p* < 0.05  versus RF and ^*δ*^*p* < 0.05  versus OVA group (Mann–Whitney *U* test).

Phylum	Class	Order	RF	OVA	OVA/C
Cyanobacteria	Chloroplast	Streptophyta	0.000 ± 0.000	0.000 ± 0.000	0.017 ± 0.010^*∗δ*^
Firmicutes	Erysipelotrichi	Erysipelotrichales	4.033 ± 1.368	0.999 ± 0.494^*∗*^	0.716 ± 0.241^*∗*^
Proteobacteria	Alphaproteobacteria	RF32	0.905 ± 0.234	0.842 ± 0.555	0.278 ± 0.065^*∗*^
Tenericutes	Mollicutes	RF39	0.083 ± 0.037	0.270 ± 0.193	2.536 ± 1.662^*∗δ*^

**Table 2:** Summary of the significant results of relative abundance of the three groups of study. Arrows indicate significant changes (*p* < 0.05) of the first group with respect to the second one.

Phylum	Class	Order	Family	Genus	Species	OVA versus RF	OVA/C versus RF	OVA/C versus OVA
Bacteroidetes	Bacteroidia	Bacteroidales				↓	↓	=
	Bacteroidia	Bacteroidales	Bacteroidaceae	*Bacteroides*	*uniformis*	=	=	↑
	Bacteroidia	Bacteroidales	Prevotellaceae	Others	Others	=	↑	↑
	Bacteroidia	Bacteroidales	Prevotellaceae	*Prevotella*	Others	=	↑	↑
	Bacteroidia	Bacteroidales	Prevotellaceae	*Prevotella*		=	↑	↑

Cyanobacteria	Chloroplast	Streptophyta				=	↑	↑

Firmicutes	Bacilli	Lactobacillales	Lactobacillaceae	*Lactobacillus*	*reuteri*	=	↑	↑
	Clostridia	Clostridiales	Lachnospiraceae	*Anaerostipes*		=	↑	↑
	Clostridia	Clostridiales	Peptostreptococcaceae	*Clostridium*	*metallolevans*	↓	↓	=
	Clostridia	Clostridiales	Ruminococcaceae	*Ruminococcus*	*flavefaciens*	=	=	↓
	Clostridia	Clostridiales	Mogibacteriaceae			=	=	↑
	Erysipelotrichi	Erysipelotrichales	Erysipelotrichaceae	Others	Others	=	=	↑
	Erysipelotrichi	Erysipelotrichales	Erysipelotrichaceae			=	↓	=
	Erysipelotrichi	Erysipelotrichales	Erysipelotrichaceae	*Allobaculum*		↓	↓	=
	Erysipelotrichi	Erysipelotrichales	Erysipelotrichaceae	*Holdemania*		=	↓	=

Proteobacteria	Alphaproteobacteria	RF32				=	↓	=

Tenericutes	Mollicutes	RF39				=	↑	↑

**Table 3:** Bacteria exclusively detected in one of the groups.

Group	Phylum	Class	Order	Family	Genus	Species
RF	Firmicutes	Bacilli	Bacillales	Staphylococcaceae	*Staphylococcus*	Others
		Bacilli	Bacillales	Staphylococcaceae	*Staphylococcus*	
		Bacilli	Bacillales	Staphylococcaceae	*Staphylococcus*	*equorum*
		Clostridia	Clostridiales	Peptostreptococcaceae	*Clostridium*	*metallolevans*
	Proteobacteria	Alphaproteobacteria	Rhodospirillales	Acetobacteraceae		
		Deltaproteobacteria	Spirobacillales			
		Gammaproteobacteria	Vibrionales	Vibrionaceae	*Vibrio*	Others
	Verrucomicrobia	Verrucomicrobiae	Verrucomicrobiales	Verrucomicrobiaceae	*Akkermansia*	*muciniphila*

OVA	Firmicutes	Bacilli	Bacillales	Bacillaceae	*Bacillus*	
		Clostridia	Clostridiales	Christensenellaceae	*Christensenella*	
	Tenericutes	Mollicutes	Anaeroplasmatales	Anaeroplasmataceae	*Anaeroplasma *	

OVA/C	Bacteroidetes	Bacteroidia	Bacteroidales	Prevotellaceae	Other	Others
		Bacteroidia	Bacteroidales	Prevotellaceae	*Prevotella*	*copri*
	Cyanobacteria	Chloroplast	Streptophyta			
	Firmicutes	Clostridia	Clostridiales	Dehalobacteriaceae		
		Clostridia	Clostridiales	Lachnospiraceae	*Anaerostipes*	
		Clostridia	Clostridiales	Veillonellaceae		
	Proteobacteria	Betaproteobacteria	Burkholderiales	Oxalobacteraceae	*Ralstonia*	
		Deltaproteobacteria	Desulfovibrionales	Desulfovibrionaceae	*Desulfovibrio*	Others
	TM7	TM7-3	CW040	F16		

**Table 4:** Bacteria present in two of the groups.

Phylum	Class	Order	Family	Genus	Species	Groups		
						RF	OVA	OVA/C
Firmicutes	Clostridia	Clostridiales	Clostridiaceae	*Clostridium*	Others	Yes	Yes	No
	Clostridia	Clostridiales	Clostridiaceae	*Clostridium*	*perfringens*	Yes	Yes	No
	Clostridia	Clostridiales	Lachnospiraceae	*Blautia*	*producta*	Yes	Yes	No
	Clostridia	Clostridiales	Lachnospiraceae	*Epulopiscium*		Yes	Yes	No
	Erysipelotrichi	Erysipelotrichales	Erysipelotrichaceae	*Coprobacillus*		Yes	Yes	No

Proteobacteria	Deltaproteobacteria	Desulfovibrionales	Desulfovibrionaceae	*Desulfovibrio*		Yes	Yes	No

Bacteroidetes	Bacteroidia	Bacteroidales	Bacteroidaceae	*Bacteroides*	*uniformis*	Yes	No	Yes
	Bacteroidia	Bacteroidales	Prevotellaceae	*Prevotella*	Others	Yes	No	Yes

Actinobacteria	Actinobacteria	Bifidobacteriales	Bifidobacteriaceae	*Bifidobacterium *	*pseudolongum*	No	Yes	Yes

**Table 5:** Correlation between intestinal IgA and microbiota. Pearson's correlation between intestinal IgA concentration and data from the absolute and relative abundance of phylum and the number of detected species of each phylum (*n* = 9). Statistical difference: ^**∗**^*p* = 0.017 (Pearson's correlation).

	Relative abundance	Absolute abundance	Number of detected species
Firmicutes	0.318	0.402	0.427
Bacteroidetes	−0.403	−0.400	−0.621
Actinobacteria	−0.375	0.253	−0.111
Proteobacteria	0.843^*∗*^	0.731	0.351
Cyanobacteria	−0.535	−0.483	−0.640
Tenericutes	−0.475	−0.440	−0.522
TM7	−0.342	−0.570	−0.243
Deferribacteres	−0.402	−0.402	−0.402
Verrucomicrobia	−0.130	0.304	−0.136

Total	0.500	0.332	0.650
